# Integration of secreted signaling molecule sensing on cell monitoring platforms: a critical review

**DOI:** 10.1007/s00216-024-05435-1

**Published:** 2024-07-24

**Authors:** Enrique Azuaje-Hualde, Juncal A. Alonso-Cabrera, Marian M. de Pancorbo, Fernando Benito-Lopez, Lourdes Basabe-Desmonts

**Affiliations:** 1 Microfluidics Cluster UPV/EHU, BIOMICs Microfluidics Group, Lascaray Research Center, University of the Basque Country UPV/EHU, Vitoria-Gasteiz, Spain; 2 BIOMICs Research Group, Lascaray Research Center, University of the Basque Country UPV/EHU, Vitoria-Gasteiz, Spain; 3 Microfluidics Cluster UPV/EHU, Analytical Microsystems & Materials for Lab-on-a-Chip (AMMa-LOAC) Group, Analytical Chemistry Department, University of the Basque Country UPV/EHU, Leioa, Spain; 4 Microfluidics Cluster UPV/EHU, Bioaraba Health Research Institute, Vitoria-Gasteiz, Spain; 5 Basque Foundation of Science, IKERBASQUE, María Díaz Haroko Kalea, 3, 48013 Bilbao, Spain

**Keywords:** Cell secretion, Microenvironments, Biosensors, Microfluidics, Monitoring

## Abstract

Monitoring cell secretion in complex microenvironments is crucial for understanding cellular behavior and advancing physiological and pathological research. While traditional cell culture methods, including organoids and spheroids, provide valuable models, real-time monitoring of cell secretion of signaling molecules remains challenging. Integrating advanced monitoring technologies into these systems often disrupts the delicate balance of the microenvironment, making it difficult to achieve sensitivity and specificity. This review explored recent strategies for integrating the monitoring of cell secretion of signaling molecules, crucial for understanding and replicating cell microenvironments, within cell culture platforms, addressing challenges such as non-adherent cell models and the focus on single-cell methodologies. We highlight advancements in biosensors, microfluidics, and three-dimensional culture methods, and discuss their potential to enhance real-time, multiplexed cell monitoring. By examining the advantages, limitations, and future prospects of these technologies, we aim to contribute to the development of integrated systems that facilitate comprehensive cell monitoring, ultimately advancing biological research and pharmaceutical development.

## Introduction

### Background

The intricate interplay between cells and their complex microenvironments in multicellular organisms has sparked a growing interest in advancing technologies for cell culture research. This has led to the development of innovative technologies that enable a more accurate emulation of cellular microenvironments, thereby facilitating a closer approximation of in vitro studies to the intricate physiological conditions [1–3]. Furthermore, these new technologies are key components in the progress of personalized medicine, which may enable the applicability of complex cell environments for drug testing, diagnosis, and therapy [4, 5].

On the one hand, a multitude of technologies has emerged with the objective of enhancing the manipulation of cell culture microenvironments, facilitating the monitoring of intricate cellular interactions. Examples include the isolation of individual cells for single-cell analysis of heterogeneous samples, the precise patterning of cells to study cell-material and cell-cell interactions, the creation of three-dimensional cultures through the utilization of innovative biomaterials, and the emulation of physiological systems on microfluidic chips. These advancements represent merely a fraction of the notable progress achieved to date [1, 6–9]. On the other side, as conventional methods for cell monitoring often are invasive or limited to end-of-assay analysis, new analytical methodologies and technologies aimed for label-free and real-time monitoring of live cell cultures. However, achieving proper integration of these two aspects within a cell culture platform significantly increases the complexity of the intended technologies hindering their widespread adoption in both clinical and research settings [10–13].

While a multitude of intracellular and extracellular cell processes can be monitored in order to comprehend cell behavior, it is crucial to recognize that the monitoring of each process present different challenges. For example, physical changes such as cell morphology, size, or motility, among others, can be easily monitored when applying optically transparent materials, like glass or clear polymers, for the optical observation of the cells [14–17]. Monitoring of internal cell processes, while more challenging due to the usual requirement of dyes, has also been regularly accomplished in a non-invasive, non-destructive way. Through the utilization of optically transparent materials that enable fluorescence or luminescence readings (e.g., glass), polymers capable of accommodating electroanalytical components (e.g., polymethyl methacrylate or polydimethylsiloxane), and the application of suitable analysis configurations, processes such as DNA transcription and replication, protein expression, enzymatic activity, behavior of organelles and microtubules, and even intracellular cation mobilization, can be monitored [18–23].

However, the monitoring of extracellular events, specifically cell secretion, presents notable technical complexities in their effective integration within cell culture platforms. The intricacy of the highly specialized sensing elements necessary for capturing, recognizing, and transducing biomolecules into signals poses a challenge to their integration within cell cultures and multifactorial microenvironments. Furthermore, localizing each sensor in close proximity to the desired secretor and achieving the necessary sensitivity for detecting extremely low concentrations of each biomolecule is a challenging task. All of this generates important obstacles on the development of integrated monitoring methods and technologies on cell cultures with complex, controlled microenvironments. This is particularly evident in the case of sensing of signaling molecules, also known as soluble factors, which play a distinctive and crucial role in shaping the cellular microenvironment and orchestrating the interplay between the living elements within it.

### Secretion of signaling molecules

**Fig. 1:**
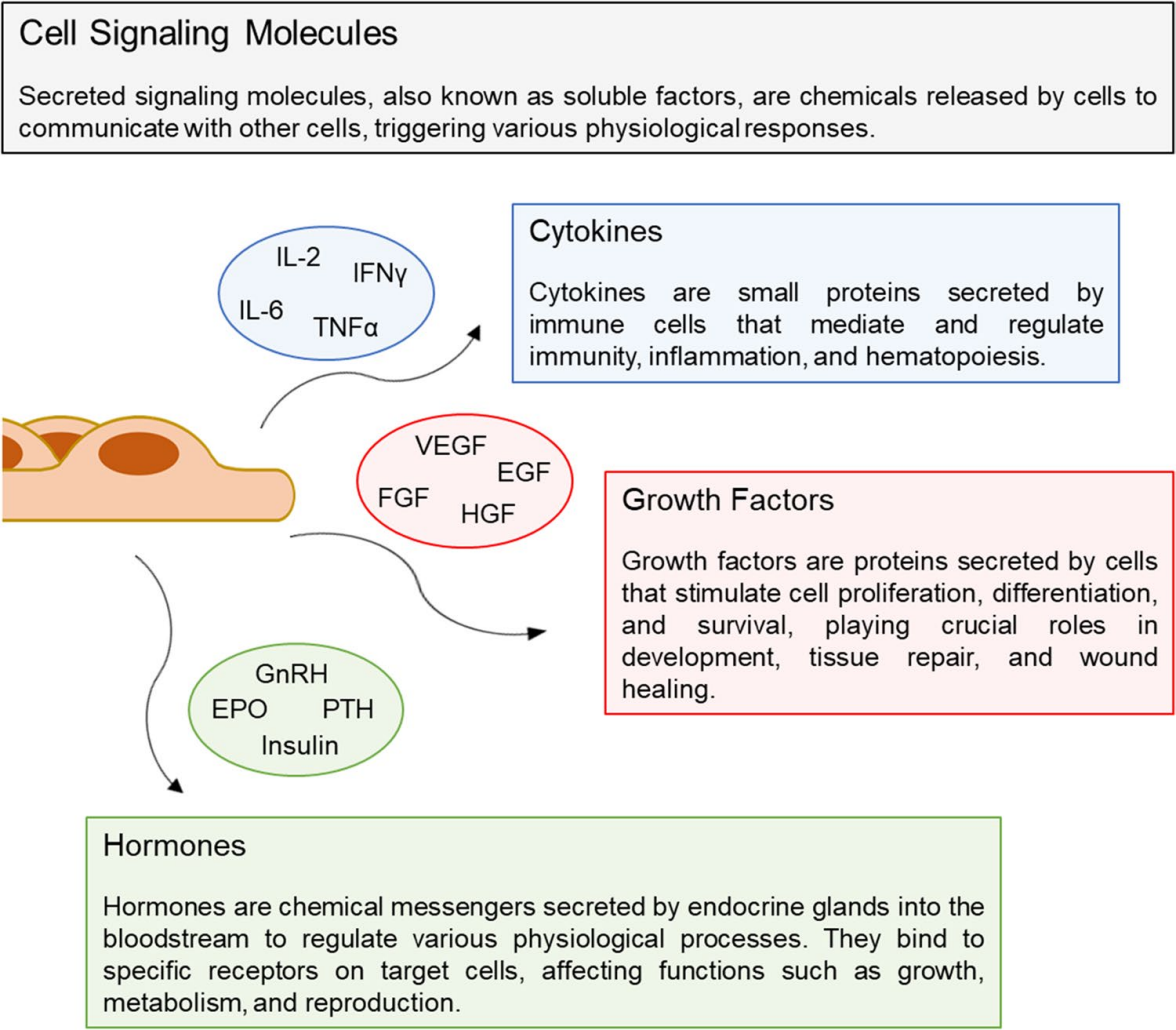
Schematic drawing of cell secretion of signaling molecules, including cytokines, growth factors, and hormones

Cells are in constant interaction with their surroundings and shape them in one form or another. Cells directly affect their microenvironment through the release of molecules into the extracellular space. For the proper functionality of a complex biological system, cells produce a massive number of biomolecules that participate at different levels in the construction, maintenance, and regulation of multicellular organisms, a process known as cell secretion. In this regard, some cell-secreted biomolecules only serve structural purposes, while others serve a regulatory role on the organism, positioning as primary signals for cell-cell communication [24]. This particular category comprises several subgroups of cell-secreted soluble factors, which mainly includes cytokines, growth factors, neurotransmitters, and hormones. These signaling biomolecules are of particular interest in the development of novel in vitro cell analytical platforms. Not only are they crucial components of the cellular microenvironment, constantly interacting with both its structural and cellular components of the microenvironment, but their monitoring is vital in order to elucidate intercellular communication in both physiological and pathological processes (Fig. 1). Furthermore, in the fields of biomedical and pharmaceutical research, signaling molecules are of key importance in discovering biomarkers and therapeutic targets essential for advancing diagnosis and treatment [25, 26].

A detailed understanding of cellular secretion of soluble factors is imperative for comprehensive studies of cell behaviors. However, detection and analysis of cell secretion presents significant challenges concerning integration within cell monitoring platforms. While the evaluation of cellular excretion of highly conserved small metabolism-related molecules such as CO_2_, lactic acid, and glucose can be accomplished through the application of established standardized assays, the integration of sensors for signaling molecules is less prevalent, primarily due to the intricate demands posed by the sensing components. These challenges include the substantial size of the biomolecules, the variations present among distinct biomolecules in diverse cellular contexts, and the low quantities in which they are secreted. Thus, this situation introduces a new set of challenges and prerequisites that must be considered when aiming to successfully implement the detection of signaling molecules and monitoring of cell secretion [27–29]. Such monitoring is highly valuable due to the extensive and informative insights it can provide into the nature and quantity of cell-secreted substances.

## The concept of biosensor for secretion monitoring

Conventional methodologies for monitoring of cell-secreted signaling molecules mostly include, but are not limited to, fluorescence- and color-based immunoassays. Conventional methods for the quantification of cell-secreted signaling molecules rely on removing cell supernatant from the culture flasks, followed by subsequent analysis using the required equipment (e.g., ELISA plate readers). This presents a series of limitations, including the impossibility to address the secretion dynamics of specific cells in complex cell cultures and the large volume required in which the analyte is diluted.

**Fig. 2:**
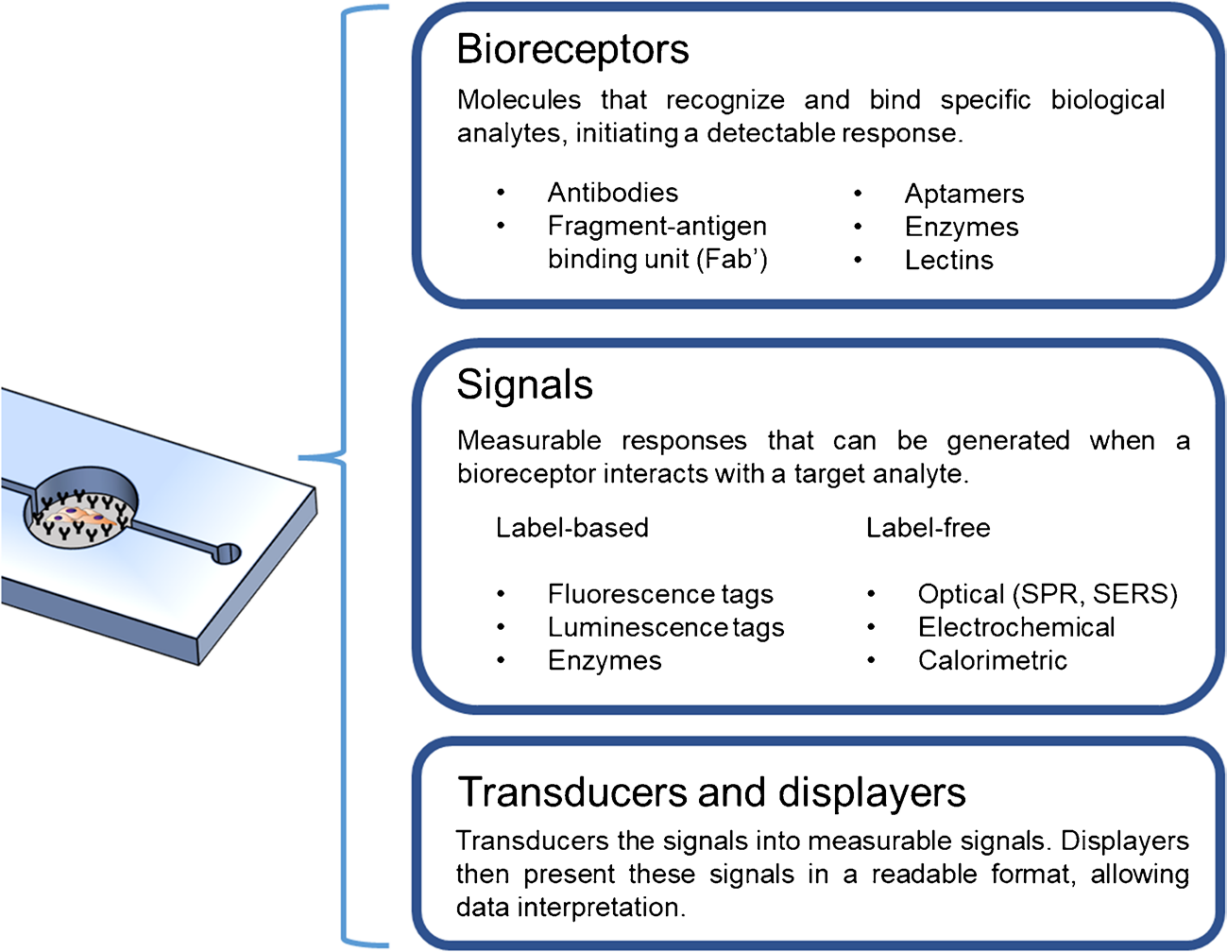
Scheme of the tool-box for cell signaling molecule biosensors, incorporating the most common choices for bioreceptors and types of signals

Taking all into account, the core of the current investigations lies in the development of biosensors that can be fully integrated within a cell culture platform, showcasing high specificity and sensitivity for the target molecule, and that neither interfere nor get affected by the complex nature of the cell microenvironment [12, 30]. Biosensors are defined as systems that enable the capture of a desired analyte, in this case a cell-secreted biomolecule, and the generation of a signal proportional to the concentration of the analyte. Equivalent detection methods, components, and equipment have been utilized in both conventional biosensing techniques and the most recent analytical devices. For the capture of the biomolecule, a bioreceptor, usually a biomolecule, is required. After capture, the production of a signal is needed to mark the presence of the successfully bound biomolecule. This can be achieved either by the interaction between the analyte and the bioreceptor itself (*label-free*) or by the addition of secondary labels. Once the signal is produced, a transducer component (which converts the signal produced into a measurable signal), a display component (which presents the user the results of the analysis), and an electronic component that binds both together are commonly required to complete the biosensor [31]. A summary of the most common components in signaling molecule biosensors can be found in Fig. 2.

In most cases, novel detection technologies still employ similar bioreceptors to those applied in conventional methodologies. For example, in the context of signaling molecules like cytokines and growth factors, antibodies are predominantly employed. Their ease of production, wide market availability, and well-established specificity still position them as the gold standard bioreceptors even in today’s landscape [32]. Other types of bioreceptors based on specific protein-enzyme and protein-lectin interactions have also been thoughtfully explored for the detection of cell secreted molecules and biomarkers. Their advantages include a reduced molecular weight when compared to antibodies, which allows a higher density of receptors in the same place, and the reduced cost of their production [31, 33, 34]. In the recent years, synthetic DNA probes such as aptamers have arisen as potential bioreceptors. They have proven to show specificity for a wide range of chemical and biochemical molecules, comparing their binding capacities to those of the antibodies. In addition to their low molecular weight, cost-effectiveness, and ease of production, they provide a variety of distinctive advantages not observed in other bioreceptors, as their sequence can be readily adjusted to augment their specificity, and they exhibit superior stability in comparison to proteins [35–37].

When it comes to the production of the signal that indicates the recognition and capture of the biomolecule, the focus has drifted into different perspectives. Directly coming from conventional cell culture monitoring technologies, colorimetric- and fluorescence-based detection systems have been extensively used in cell secretion biosensors. The easiness in differentiating between the presence and the intensities of the signals that can be related to the different quantities of the biomolecules allows their simple detection and quantification [38, 39]. Furthermore, advancements in single-molecule microscopy and Förster resonance energy transfer (FRET) systems, although not yet widely impacting the area of cell secretion monitoring, still position fluorescence-based assays to achieve the highest sensitivity in protein quantification [40–42].

In the lookout for label-free biosensors, new methodologies have arisen. Optical label-free detection has been widely explored, which includes but is not limited to surface plasmon resonance (SPR) and surface-enhanced Raman spectroscopy (SERS). These methods are based on the observation of specific changes of physical phenomena such as light scattering or molecular vibration, which can be correlated with the presence and concentration of an analyte [43, 44]. Electrochemical detection has also been explored for cell secretion biosensing, being based on the transduction of the biochemical event into an electrical signal, which can be a change in current, voltage, or impedance, among others [30, 45]. Finally, colorimetric-based and magnetic-based detection methods have been proposed and explored to a lesser degree [46].

Depending on the expected outcomes, combinations of the previously described bioreceptors and signals may be utilized, as none of them singularly represents an ideal solution. While antibodies are a reliable choice as bioreceptors, particularly in platforms for multiplex analysis of a wide variety of cell-secreted biomolecules, synthetic DNA probes (such as DNAzymes, molecular beacon probes, and structure switching signaling aptamers) may be more suitable for label-free monitoring. These probes have specific three-dimensional conformations that undergo structural changes upon recognizing the target molecule, enabling them to produce a quantifiable signal without the need for a secondary label [47–49]. Nevertheless, when pursuing label-free monitoring in a general context, electrochemical and optical biosensing emerge as superior methodologies. This is because, by definition, they enable the label-free identification of secreted biomolecules by solely measuring the capture of the analyte by the sensor. Yet, these methods are not without their inherent constraints, such as the challenge of simultaneously detecting multiple analytes coming from a complex sample within a limited analysis space [50, 51]. This may explain why fluorescence-based detection methods remain the most suitable detection strategies to incorporate into a multifactorial cell culture platform, as they are still the best option for the multiplex analysis of biological samples.

In all, a consensus has not yet been reached on which type of biosensor is most suitable for use in a wide range of scenarios. Further discussion is necessary to compare the different strategies and determine their respective advantages and limitations in various experimental contexts.

## Platforms for cell culture secretion monitoring

In recent years, there has been a substantial upsurge in interest in the development of systems for monitoring cell cultures. This could be attributed to a series of factors. On the one hand, the exploration of microfluidics and lab-on-a-chip devices has allowed the generation of intricate systems for cell culturing devices. The ability to create distinct and separated compartments for cell culture and biosensing, each tailored with their specific chemical and biochemical functionalizations, along with the capacity to construct intricate fluidic networks that interconnect these sections represents just a subset of the advantages offered by these systems [51–54]. On the other hand, advances on materials and biomaterials research has enabled both the development of suitable substrates for the generation of complex microenvironments and the fabrication of novel biosensors. The arising of smart, functional, and biocompatible materials in the recent years has allowed to speed up the development of multifunctional platforms for cell culture [55–57]. While most of what has been achieved so far offers a promising look on what can be accomplished in the near future, these new platforms are still on very early stages of development and can only be defined as proof-of-concept.

Ideally, a complete integrated platform should combine the culture of cells under a controlled microenvironment and the monitoring of cell secretion within the same device, independently of the applied biosensing method. However, the absence of a standardized definition for what constitutes a platform that truly integrates cell culture and secretion, as well as the specific components that should be included within such a platform, can be primarily attributed to the early developmental stage of this research area. Consequently, what has been described as “integrated” up to this point exhibits differing levels of implementation concerning cell culture and secretion monitoring within the same system.

Taking all into account, we have focused our literature survey on recent cell monitoring platforms that combine the capture and maintenance of cells, whether through regular 2D cultures, microfluidic-based cell trapping, or the incorporation of 3D cultures, with the integration of bioreceptors for capturing signaling molecules within a single device. This includes both devices that require further extraction of the bioreceptors for analysis, usually employing barcode systems and microbeads [58–62] and those that enable direct analysis of secretion within the same device. Table 1 lists the publications considered within these parameters, detailing the biological and sensing characteristics.

**Table 1:** List of cell monitoring platforms that combine the capture and maintenance of cells. ^†^High-throughput analysis (hundreds to thousands of individual cell events); ^††^label-free measurements

Measurement type	Cell type measured	Co-culture	Type of culture	Single cell	Biosensor location	Localized sensing	Biomolecules	Bioreceptor	LOD	Equipment	Author and year
Fluorescence	Monocytes	Cells assessed individually	Uncontrolled	No	Biosensing compartment	No	TNF-β, INF-γ, IL-1, and IL-8	Antibody	-	Plate reader	Kongsuphol et al. 2016 [60]
Fluorescence	Macrophages	Cells assessed individually	Cell trap	No	Biosensing compartment	No	TNF	Antibody	5.86 ng mL^−1^	Fluorescence microscope	Kaestly et al. 2017 [63]
Fluorescence	Hepatocytes	Cells assessed individually	Cell trap	No	Biosensing compartment	No	HGF and TGF	Antibody	0.5 and 0.9 ng mL^−1^	Fluorescence microscope	Son et al. 2017 [91]
Fluorescence	Peripheral blood mononuclear cells (PBMCs)	Cells assessed individually	†Cell trap	No	Biosensing compartment	No	IL-6, IL-8, and TNF-α	Antibody	0.02 ng mL^−1^	Fluorescence microscope	Cui et al. 2018 [64]
Fluorescence	Adenocarcinoma cells (MCF7 y MDA)	Cells assessed individually	†Droplet	Yes	Droplet	Yes	IL-6, IL-8, and MCP-1	Antibody	0.9, 0.7 and 0.4 ng mL^−1^	Fluorescence microscope	Hsu et al. 2018 [87]
Fluorescence	Tumor-derived endothelial cells; endothelial cells	Cells assessed co-cultured	Cell trap	No	Biosensing compartment	No	IL-8	Antibody	0.02 ng mL^−1^ (semiquantitative)	Confocal microscope	Zhang et al. 2018 [65]
Fluorescence	Microglial cells	Cells assessed individually	Uncontrolled	No	Cell surface	Yes	IL-6	Antibody	0.001 ng mL^−1^	Fluorescence microscope	Liu et al. 2019 [83]
Fluorescence	Macrophages	Cells assessed individually	†Cell trap	No	Biosensing compartment	Yes	TNF, CCL2, CCL3, and CCL5	Antibody	0.05 ng mL^−1^	Confocal microscope	Ramji et al. 2019 [66]
Fluorescence	Monocytes (THP1)	Cells assessed individually	†Holes	Yes	Underneath	Yes	IL-1, IL-6, IL-8, MIF, MCP-1, and TNF	Antibody	0.009, 0.003, 0.007, 0.004, 0.001 and 0.03 ng mL^−1^	Fluorescence microscope	Abdullah et al. 2019 [77]
Fluorescence	NK92 cells	Cells assessed co-cultured with leukemia cells	†Droplet	Yes	Droplet	Yes	IFN-γ	Antibody	66.4 ng mL^−1^	Fluorescence microscope	Antona et al. 2020 [85]
Fluorescence	NK-92 MI cells	Cells assessed co-cultured with lymphoblasts (K562)	†Droplet	Yes	Droplet	Yes	IFN-γ	Antibody	Qualitative	Fluorescence microscope	Yuan et al. 2020 [86]
Fluorescence	Lymphocytes	Cells assessed co-cultured with PC3, HeLa and Raji cells	†Holes	Yes	Around cells	Yes	IFN-γ	Antibody	0.16 ng mL^−1^	Fluorescence microscope	Zhou et al. 2020 [74]
Fluorescence	Circulating tumor cells	Cells assessed individually	†Cell trap	Yes	Around cells	Yes	G-CSF	Antibody	1.5 ng mL^−1^	Fluorescence microscope	Armbrecht et al. 2020 [70]
Fluorescence	Monocytes (THP-1); PBMCs	Cells assessed individually	†Cell trap	No	Biosensing compartment	No	IL-8 and TNF-α	Antibody	2.5 and 2 ng mL^−1^	Fluorescence microscope	R-Moncayo et al. 2020 [67]
Fluorescence	Neonatal dermal fibroblasts	Cells assessed individually	Uncontrolled	No	Cell surface	No	HGF	Antibody	0.1 and 0.034 ng mL^−1^	Flow cytometry–based lasers	Neel et al. 2020 [82]
Fluorescence	Macrophages	Cells assessed co-cultured with carcinoma cells	Holes	No	On top of the cells	Yes	MIP-1β, IL-10, IL-8, MCP-1, IL-6, and TNF-α	Antibody	Qualitative	Laser scanner	Linmei et al. 2021 [61]
Fluorescence	Astrocytes	Cells assessed individually	†Holes	Yes	On top of the cells	Yes	GM-CSF, IL-12, IL-1β, IL-2, IL-4, IL-6, IL-8, IL-17a, MCP-1	Antibody	0.002, 0.002, 0.002, 0.003, 0.001, 0.001, 0.0002, 0.002 and 0.004 ng mL^−1^	Laser scanner	Shao et al. 2021 [59]
Fluorescence	Monocytes	Cells assessed individually	†Droplet	Yes	Droplet	Yes	TNF-α	Antibody	-	Fluorescence microscope	Llitjos et al. 2021 [94]
Fluorescence	Astrocytes	Cells assessed individually	Uncontrolled	Yes	Underneath	Yes	TNF-α and IL-1β	Antibody	-	Fluorescence microscope	Khodadadei et al. 2021 [80]
Fluorescence	Adenocarcinoma lymphoblasts (K562); leukemia cells (293 T); neuroblastoma cells	Cells assessed individually	†Holes	Yes	On top of the cells	Yes	IL-6 and IL-8	Antibody	0.004 and 0.0005 ng mL^−1^	Laser scanner	Wang et al. 2022 [62]
Fluorescence	Monocytes (THP-1)	Cells assessed individually	Cell trap	No	Biosensing compartment	No	TNF-α, IL-1β, IL-6, and IL-10	Antibody	0.025 ng mL^−1^	Plate reader	Ramadan et al. 2022 [58]
Fluorescence	Monocytes (THP-1)	Cells assessed co-cultured with epithelial cells carcinoma cells	Cell trap	No	Biosensing compartment	No	TNF and IL-12p70	Antibody	0.004 and 0.003 ng mL^−1^	Fluorescence microscope	Cui et al. 2022 [95]
Fluorescence	Blood-derived monocytes	Cells assessed individually	†Cell trap	Yes	Around cells	Yes	IL-8	Antibody	0.011 ng mL^−1^	Fluorescence microscope	Cedillo-Alcantar et al. 2023 [72]
Fluorescence	Macrophages	Cells assessed individually	†Cell trap	Yes	Around cells	No	IL-10, VEGF, and TNF-α	Antibody	1.5, 0.5 and 0.2 ng mL^−1^	Fluorescence microscope	Dietsche et al. 2023 [73]
††Nanoplasmonics	Adenocarcinoma cells (HeLa)	Cells assessed individually	Uncontrolled	No	Biosensing compartment	No	VEGF	Antibody	0.145 ng mL^−1^	Spectrometer	Li et al. 2017 [92]
††SPR	Adipocyte; macrophages	Cells assessed co-cultured	†Adipose-tissue-on-chip	No	Around cells	No	IL-6, IL-10, and TNF-α	Antibody	0.02 ng mL^−1^	Dark-field microscope	Zhu et al. 2018 [75]
††Nanoplasmonics	Lymphoma cell (EL4)	Cells assessed individually	Cell trap	Yes	Underneath	Yes	IL-2	Antibody	0.039 ng mL^−1^	Spectrometer	Li et al. 2018 [79]
††SPR	Adenocarcinoma cells (MDA); leukemia cell (HL-60)	Cells assessed individually	†Droplet	Yes	Droplet	Yes	IL-8 and VEGF	Antibody	7.2 and 6.39 ng mL^−1^	Spectrometer	Wei et al. 2019 [88]
††SPR	Jurkat T cells	Cells assessed individually	†Holes	Yes	On top of the cells	Yes	IL-6	Antibody	10 ng mL^−1^	Spectrometer	Zhu et al. 2020 [89]
††SPR	Pancreatic islets	Cells assessed individually	Islet-on-a-chip	No	Biosensing compartment	No	Insulin	Antibody	850 ng mL^−1^	Spectrometer	Ortega et al. 2021 [69]
††Nanoplasmonics	Lymphoma cell (EL4)	Cells assessed individually	†Holes	Yes	Underneath	Yes	IL-2	Antibody	-	Fluorescence microscope spectrometer	Yen-Cheng et al. 2022 [81]
††Plasmonic	T lymphoblast	Cells assessed individually	†Holes	Yes	Underneath	Yes	IL-2	Antibody	-	Inverted microscope + collimated near-infrared LED	Ansaryan et al. 2023 [76]
††SERS	Adenocarcinoma cells (MCF7) endothelial cells (HUVECs)	Cells assessed individually	Scaffold	No	Biosensing compartment	No	VEGF	Antibody-aptamer	0.1 ng mL^−1^	Raman spectrometer	Qian et al. 2020 [68]
††Raman microspectroscopy	Pancreatic pseudo-islets (endo c-bh3 cell)	Cells assessed individually	Pancreas-on-a-chip	No	In the medium	No	Insulin	-	Qualitative	Raman microscope	Zbinden et al. 2020 [99]
††SERS	Adipose-derived mesenchymal stem cells	Cells assessed individually	Scaffold	No	On top of the cells	No	OC (ALP and FN)	Antibody	0.01 ng mL^−1^	Raman spectrometer	Ko et al. 2021 [90]
††SERS	Adenocarcinoma (MCF-7, SGC, and T24)	Cells assessed individually	†Droplet	Yes	Cell surface	Yes	VEGF	Antibody	Qualitative	Confocal Raman microscope; fluorescence microscope	Cong et al. 2022 [84]
††Electrochemical	Hepatocytes and stellate cells	Cells assessed individually and co-cultured	Liver injury-on-a-chip	No	Biosensing compartment	No	TGF-β1	Aptamer	1 ng mL^−1^	Potentiostat	Zhou et al. 2015 [71]
††Electrochemical	Neuroblastoma cells; keratinocytes	Cells assessed individually	Uncontrolled	No	Biosensing compartment	No	PTHLH	Antibody	55 ng mL^−1^	Potentiostat	E-Muñiz et al. 2018 [78]
††Electrochemical	PBMCs	Cells assessed individually	Uncontrolled	No	Underneath	No	IFN-γ	Aptamer	0.006 ng mL^−1^	Chrono-amperometry	Liu et al. 2019 [93]

### Integration of signaling molecule sensing on cell monitoring platforms

Cell monitoring platforms that integrate sensing for secreted signaling molecules can be categorized into two main groups based on the placement of the bioreceptors: those where bioreceptors are placed in a biosensing compartment independent from the cells, and those where bioreceptors are placed in direct proximity to the cells (see Table 1, column 6 “Bioreceptors”).

**Fig. 3:**
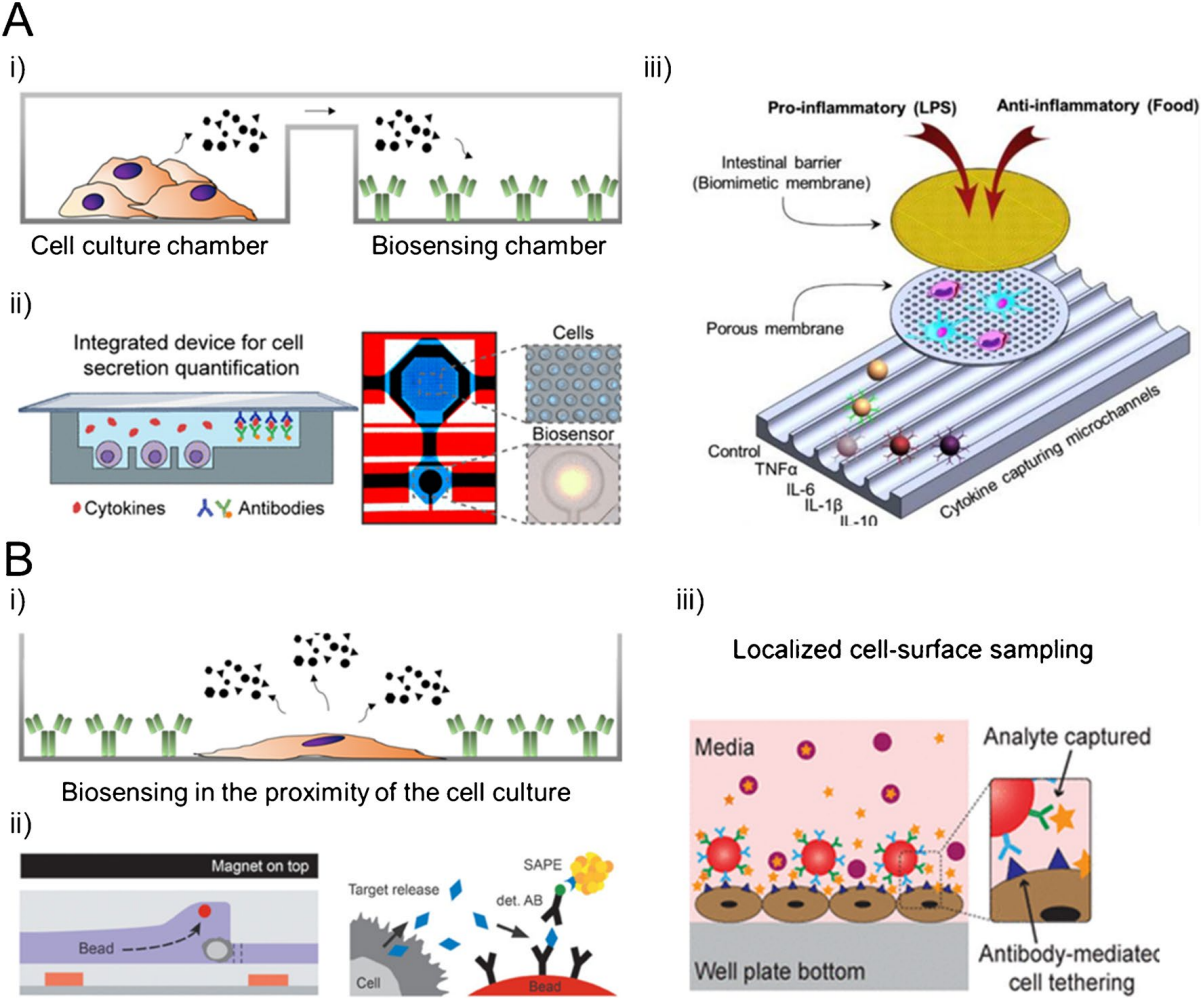
Strategies followed for the monitoring of the secretion of signaling molecules of a cell culture. **A** Transport of the secreted biomolecules from the culture chamber to the biosensing chamber within a microfluidic device. (i) A schematic drawing of the devices. (ii) and (iii) Examples of real devices, adapted from Rodriguez-Moncayo et al. [67], with the permission of ACS publications, and from van Neel et al. [82] with the permission of ACS publications. **B** Controlled placement of the biosensors in the proximity to the cell culture. (i) A schematic drawing of the devices. (ii) and (iii) Examples of real devices, adapted from Armbrecht et al. [70], with the permission of Wiley, and from Ramadan et al. [58] with the permission of AIP publishing

The first approach employs microfluidics to create complex systems with fluidic networks that transport secretions from each cell culture compartment to a dedicated biosensing compartment [58, 63–67, 67–73] (Fig. 3A). These compartments can be either fully integrated into a single device or exist as modular components that can be interconnected.

The second strategy relies on directly detecting secreted biomolecules from each cell event by placing bioreceptors in close proximity to the cells, often utilizing particles or features like barcodes. Integrating both cell culture and biosensing within a single compartment enables immediate capture of cell secretion and its precise correlation with the secretory cell. The bioreceptors or biosensors can be positioned around the cells [70, 74–76], underneath the cells [59, 77–81], on the cell surface [82, 83], in the medium [60, 64, 85–88], and on top of the cells [61, 62, 89, 90] (Fig. 3B).

Both strategies offer their own set of advantages and limitations. Placing bioreceptors in close proximity to the cells enables monitoring cell secretion with a spatial resolution unattainable by conventional methods, enhancing the ability to correlate cell secretion with individual cell events. However, this advantage comes with increased complexity in modulating substrates to accommodate both cellular and sensing elements, and it is more restrictive when combining cell secretion analysis with the other optical methods for cell monitoring. This complexity is particularly evident when unconventional materials are required as part of the system, such as polydimethylsiloxane (PDMS) for most microfluidic devices and gold layers for SPR-based sensing. On the other hand, generating individual but interconnected compartments facilitates fabrication, enabling adaptation of each compartment to its specific requirements. This not only allows for proper cell culture without the burdens that arise from the sensing requirements, both from a chemical and material point of view, but also enables easy integration of conventional assays such as immunocytochemistry. However, this approach sacrifices the spatial resolution achieved by the first strategy. In summary, one strategy does not surpass the other; each approach should be considered based on the type of analysis desired.

### Main biological models explored

Most platforms developed to date have focused on exploring well-known secretion models to test and validate the technologies. This approach has led to a limited catalog of signaling biomolecules applied in cell sensing and a clear trend in the biological models utilized. Notably, immune cell-related models, often coupled with cytokine sensing, are the most extensively explored (Table 1, column 2 “Cell type measured”, column 8 “Biomolecules”). Interleukins (IL)-2, 4, 6, and 8; tumor necrosis factor (TNF); and interferon-γ (IFN) are some of the secreted cytokines whose detection and quantification have been incorporated in the most recent cell culture platforms [58–64, 70, 72, 73, 75–82, 85, 86, 89–95]. There are several reasons for researchers to use this particular model. Firstly, cytokine secretion models are well known and have been thoughtfully studied over the past years, presenting a broader window of standardized detection methodologies that can be adapted to new platforms, which facilitates the development and their validation of novel biosensing techniques. Secondly, cytokines are widely used as biomarkers and reporters to address pathological conditions, giving to the resulting product an excellent potential to be applied in clinical practice [96, 97]. Finally, the type of cell cultures used to generate these models have been mostly based on cells derived from the immune system [58, 60, 63, 64, 66, 67, 72, 73, 75–77, 85, 86, 89, 93–95], which are notably easier to integrate into a platform when compared to other cell types. This ease of integration is largely attributed to their non-adherent or transient nature, which in turn requires a less intricate microenvironment for their proper cultivation, especially in contrast to cell types derived from complex physiological microenvironments [98]. Therefore, it is easier to implement the replication of their microenvironment inside a platform.

A second model also widely used is the monitoring of growth factor secretion, such as the vascular endothelial growth factor (VEGF) and the hepatocyte growth factor (HGF) [60, 68, 70, 82, 88, 92]. These well-established models also present a huge clinical potential due to their regulatory actions and relation with pathological conditions. Another example of a secreted molecule that has been reported is a hormone like insulin [69, 99].

While the models preferred for developing new technologies represent only a fraction of the many different types of microenvironments, incorporating cytokine sensing in these novel platforms has enabled cell monitoring assays previously unreachable with conventional technologies. For example, there have been remarkable studies on the biochemical cross-talk between cancer cells and their physiological and immunological microenvironments. This has been achieved through the co-encapsulation of single cancer cells with, for instance, lymphocytes and endothelial cells. These novel approaches have the potential to unveil new knowledge about important, yet-to-be-understood processes in cancer research, such as immunoediting [68, 70, 74, 85].

### Novel approaches: multiplexing, label-free, and high-throughput

The need to study cell behavior while emulating the cellular microenvironment has driven the pursuit of novel data acquisition approaches, improving and expanding the type of analysis performed through conventional methods. In those regards, the current goal lies in simultaneously detecting multiple biomarkers in real-time across thousands of scenarios. Therefore, advances in this field have focused primarily on three areas: high-throughput analysis of numerous replicas for robust data acquisition, multiplex analysis of a wide range of cell-secreted signaling molecules from a single secretor, and label-free and spatiotemporal resolved analysis of cell secretion on live cells.

Whereas high-throughput analysis has been uniformly adopted in the microtechnologies developed, usually achieved through patterning or capturing hundreds to thousands of individual cell events on a single platform, the implementation of multiplex detection and label-free analysis has taken different approaches with contrasting perspectives. It has been observed that the basis for developing either multiplex or label-free cell monitoring systems usually limits the implementation of the other. This contention arises from the types of signals used to achieve the desired outcomes. Although platforms aimed at both types of analysis share common aspects, such as appropriate limits of detection for cell culture applications (in the pg mL^−1^ to ng mL^−1^ range) and the use of antibodies as the most common bioreceptors (see Table 1, column 9 “Bioreceptor”), there is a clear divide in the approaches taken based on the desired type of analysis.

**Fig. 4:**
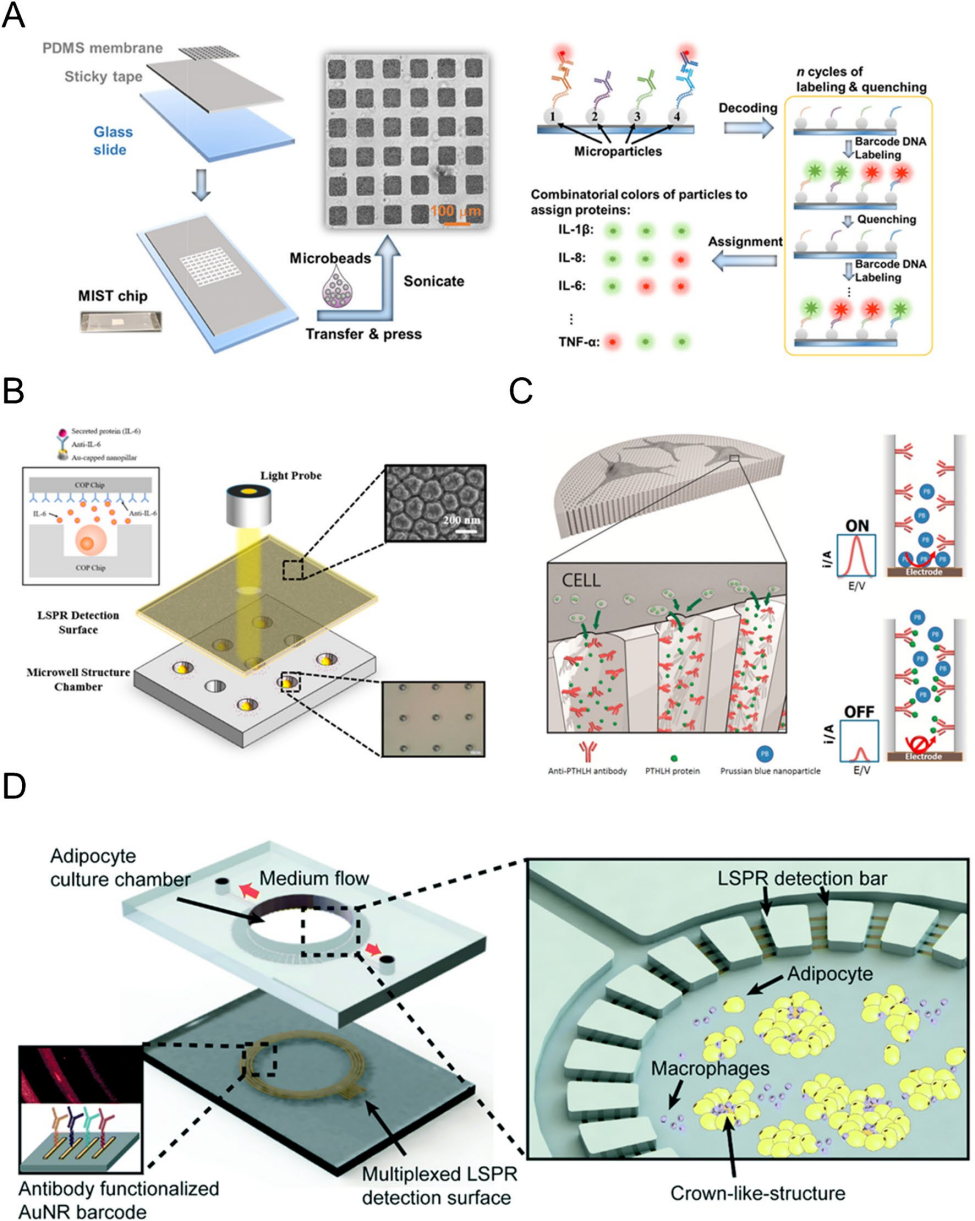
Systems for multiplex and label-free monitoring of cell secretion. **A** Microbead-based multiplex array for the detection of single-cell secretion of cytokines. Adapted from Abdullah et al. [77] with the permission of the American Chemical Society. **B** Microwell structure for the detection of cell secreted cytokine using localized SPR. Adapted from Zhu et al. [89] **C** Platform for the localized electrochemical detection of parathyroid hormone–like hormone secreted from a cell culture. Adapted from Escosura-Muñiz et al. [78] with the permission of Elsevier. **D** Optofluidic chip for the SPR-based multiplex detection of cytokines secreted from an adipocyte culture. Adapted from Zhu et al. [75] with the permission of the Royal Society of Chemistry

In multiplex analysis, which focuses on discerning a high quantity of distinct secretory events to unveil the secretory profile of a cell, the technologies used typically rely on immunofluorescence assays (Fig. 4A) [58–62, 64, 66, 67, 73, 77, 80, 87, 91, 95]. Unlike other techniques, fluorescence-based assays enable the simultaneous detection of multiple signals by using different labels. This approach provides the required sensitivity, showcasing the lowest limits of detection found in the literature (see Table 1, column 10 “LOD”), and allows independent detection of biomolecules, facilitating the identification of 5 to 9 different secreted signaling molecules from a single cell event [59, 77]. The widespread use of these techniques however limits their implementation on real-time assays due to the end-of-assay nature of the systems.

In contrast, most label-free systems, which focus on achieving spatiotemporal-resolved analysis of individual secretory events, have relied on plasmon-based monitoring techniques such as SPR or SERS (Fig. 4B) [68, 69, 75, 84, 89, 90]. These approaches can easily detect the presence of a desired analyte without the need for secondary labels, due to the specific changes in resonant conditions that occur when biomolecules approach metallic plasmonic structures functionalized with specific biomolecules for analyte capture. SPR-based analysis of cell secretion has demonstrated excellent outcomes in studying single-cell secretion dynamics, allowing for simultaneous analysis of the spatial diffusion of cell secretions and the real-time profiling of secretion rates [76]. However, these strategies limit the implementation of multiplex analysis due to the exponential complexity involved in discerning multiple biomolecules, and thus multiple signals, through plasmon-based systems.

While platforms that enable both multiplex and label-free analysis are still uncommon, advances in other signal detection methods, such as electrochemical outputs, and the development of novel bioreceptors, such as aptamers, could reshape the current landscape. Developing novel fluorescence or luminescence-based systems for real-time monitoring could solve the challenge of combining multiplexing with real-time monitoring [64, 80]. Furthermore, integrating multiple detection systems within a single platform can achieve these outcomes. For instance, Zhu et al. [75] demonstrated in their work that combining SPR with electrochemical sensors allows for multiplexing and label-free monitoring (Fig. 4C). They developed an optofluidic device that enabled the multiplex detection of secreted pro- and anti-inflammatory cytokines (IL-4, IL-6, IL-10, and TNF-α) from an adipose tissue culture using localized SPR.

### Monitoring of secretion on complex cell culture microenvironments

**Fig. 5:**
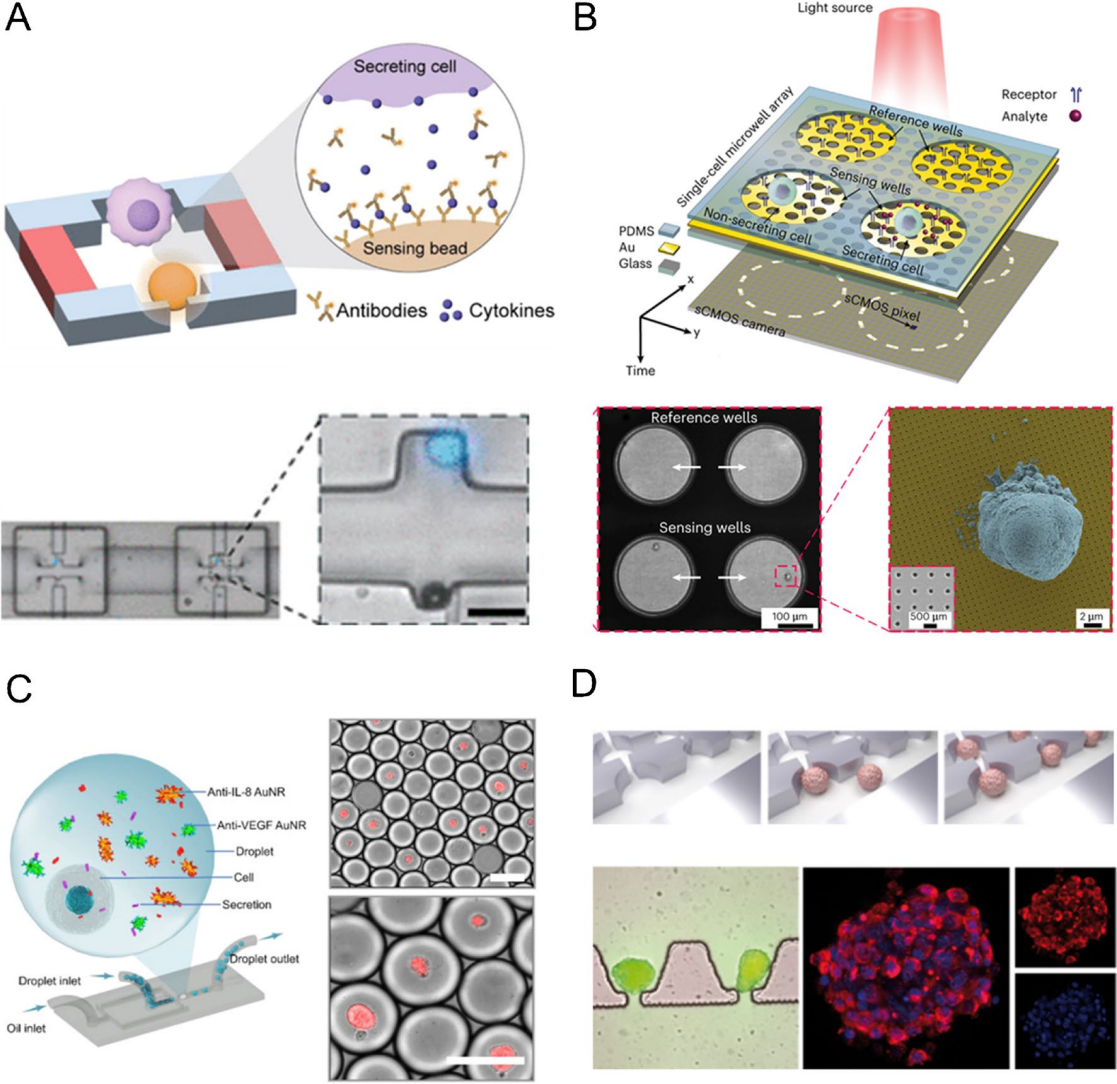
Systems for controlling cell positioning, sorting on 2D and 3D cultures. **A** Microfluidics-based sorting of single cells in microchambers. Adapted from Cedillo-Alcantar et al. [72] with the permission of the ASC publications. **B** Sorting of single cells in microwell arrays. Adapted from Ansaryan et al. [76] with the permission of the Springer Nature. **C** Droplet-based single-cell sorting. Adapted from Wei et al. [88] with the permission of Elsevier. **D** Organ-on-a-chip sorting of pancreas islets. Adapted from Zbinden et al. [99] with the permission of Elsevier

Earlier platforms for monitoring cell secretion of signaling molecules focused on incorporating biosensors either in the vicinity of the cells or through the interconnection of the culture and sensing chambers, without precise control over cell positioning or the complexity of the microenvironment [60, 78, 80, 82, 83, 92, 93]. As more integrated platforms were gradually implemented, different approaches emerged to improve control over cell interactions and enhance microenvironmental complexity. This was made possible through the implementation of microfabrication and microfluidic technologies, which enabled better biochemical adaptation of surfaces, the ability to control cell placement in 2D, droplets, or microfluidic devices, as well as the generation of complex 3D structures more suitable for cell culture (Fig. 5) [58, 59, 61–77, 79, 81, 84–91, 94, 95, 99].

Among the different methods taken, the first consists on controlling the positioning of either single cell or cell colonies through fluidic sorting [63–68, 70–73, 79, 91, 95]. This can be achieved by trapping and sorting them by making use of the architecture of microfluidics devices, which can span from a conformation of cavities in a PDMS chip [59] to a more intricate network of microchambers [72]. This can also be combined with surface functionalization using materials that resembles the desired cell microenvironment, such as collagen, prior to cell seeding [63, 71, 92]. The second method consists on the encapsulation or entrapment of cells within a 3D matrix. In this case, the most used strategy is the generation of droplets in which the cells are encapsulated (either single cells or cell in co-cultures), in which the bioreceptors or biosensors are localized in the matrix of the droplet [85–87]. Finally, the last approach consists on the use of biomimetic scaffolds for the 3D culture of cells, placed in close proximity to the biosensors [68, 69, 90].

In light of this, there is a clear trend towards controlling cell positioning, with less emphasis on managing the physical and biochemical interactions found in microenvironments, as more than half of the platforms reviewed focus in single cell studies (Table 1, column 5 “Single cell”) [59, 62, 70, 72–74, 76, 77, 79–81, 89]. This is due to the increasing interest in the study of the cell behavior in a single-cell level, allowing an easy implementation of high-throughput analysis and the investigation of cellular heterogeneity. The next most developed platforms are the ones based on droplet generation in which one cell or two cells of different cell types (usually being one of them from the immune system line) are entrapped alongside the biosensor [84–86, 88, 94].

Overall, there have been only a few significant advancements in platforms designed to monitor cell secretion in highly structured microenvironments with one or more cell types. This scarcity of progress can be ascribed to, as previously mentioned, the prevalence of non-adherent cells as models for secretion, which do not represent the most common type of cell microenvironment present in complex organisms such as mammals and humans, and the focus on single-cell monitoring, a non-representative model for physiological and pathological conditions as cells are not usually found in an isolated state in their physiological microenvironments.

The lack of integration of biosensor for secretion monitoring is very relevant when looking at the platforms that try to replicate controlled physiological conditions, such as organoids and organ-on-chip devices [100–102]. While sensors aimed to monitor the physical and metabolic processes have been developed, sensors for cell secretion, especially when it comes to signaling molecules, are rarely integrated. Considering all this, there is a clear gap in exploring new platforms that truly integrate complex microenvironments and cell secretion monitoring. It is evident, from the lack of literature, that the development of platforms to monitor secreted signaling biomolecules in complex microenvironments is limited. This outcome is expected given the quick progress made in expanding the landscape of cell cultures. As the complexity of both for the biological models utilized and for the technologies required to achieve these models increase, established integrated sensing strategies become inadequate, particularly in a research area lacking standardization of methods even for the most basic models.

Nevertheless, a few systems have arisen aiming to combine organ-on-chip technology with integrated cell secretion monitoring processes (Table 1, column 4 “Type of culture”). For instance, Zhou et al. [71] developed a liver injury-on-chip microfluidic device that enabled the co-culture of hepatocytes and stellate cells for the electrochemical monitoring of transforming growth factor (TGF) secretion, using aptamers as bioreceptors. A bit later, Zhu et al. [75] developed a tissue-on-chip that replicated an obese adipose tissue biomimetic microenvironment for the detection of cytokines secretion (IL-6, IL-10, and TNF) using SPR barcodes. Recently, Zbinden et al. [99] developed a human pancreas-on-a-chip, utilizing 3D pancreas islets, for the monitoring of the endocrine function, specifically for the non-invasive real-time monitoring of insulin secretion using Raman microspectroscopy. Ortega et al. [69] developed a biomimetic pancreatic-islets-on-chip for the label-free and in situ monitoring of insulin secretion through localized SPR.

## Conclusions and future perspectives

In this critical review, we have examined the current landscape of cell secretion monitoring within complex microenvironments, highlighting both the progress made and the significant challenges that remain. Every year, the quantity and complexity of platforms developed to monitor cell secretion increase, indicating promising advancements in the near future. While there is no consensus yet on the best approach to developing these technologies, prominent advancements in biosensing, materials, microfabrication, microfluidics, and biological sciences continuously provide new possibilities.

Over the past decade, there has been a notable trend in developing platforms for monitoring cell secretion of signaling molecules, with a primary emphasis on single-cell analysis of biological systems tied to immunological processes. These models are excellent both from the perspective of their easier implementation in a research area that is still in the proof-of-concept stage, and the relevant information that can be obtained from them. However, they are far from being representative of how most microenvironments function. Researchers agree on the need for technologies that enable cell secretion monitoring of more complex biological models, especially those that take into account the importance of cell-cell contact and the physical and chemical characteristics of the extracellular matrix [69, 71, 75, 76, 99]. As such, a push towards integrating biosensors within more complex cell culture methods is expected, particularly with the rise of organ-on-a-chip devices. This integration, however, should be accompanied by active and specialized research on signaling molecule sensing strategies within a more complex microenvironment. Especially with the advent of 3D cultures, it has become imperative to develop yet-to-be-seen sensing methods that can detect secretions within the entire three-dimensional space and correlate these secretory events to individual cells within the complex 3D matrices.

Regarding what biosensing methods should be applied, a consensus has not been reached and this is not expected to change in the near future. In those regards, antibodies are still the most prevalent choice for bioreceptors, and while new research is emerging in the area of aptamers and nanobodies, the commercial availability and reliability of antibodies still make them the desirable choice in all areas [58–67, 69, 70, 72–92, 94, 95]. In terms of the debate between label-based and label-free methods, especially when it comes to conventional immunoassays versus plasmon-based analysis, the path forward remains unclear and fluorescence-based methods stay the most prevalent choice due to the ease they offer both to end-users and novel researchers developing cell monitoring platforms. This preference stems from their standardization in traditional cell analytical assays, which simplifies their use. Additionally, fluorescence-based methods offer the significant advantage of straightforward multiplexing. Nevertheless, plasmon-based systems, despite their limitations, are rapidly advancing and achieving new types of analysis never accomplished before, such as the real-time monitoring of secretion diffusion from a single cell. Considering this, it is more than possible that, just like other traditional cell monitoring methods such as flow cytometry and immunocytochemistry co-exist, both strategies will be necessary for a full understanding of complex microenvironments. This is especially true with the rise of proteomics research, advances in mass spectrometry for detecting signaling molecules, and the growing interest in studying secreted vesicles. Although these have not yet been integrated into in vitro systems, they will add complexity to the current landscape [103–105]. Therefore, it is recommended that future research focuses not only on expanding the current strategies, but also on allowing the combination of different approaches, for instance, the integration of both fluorescence measurements and SPR analysis within a single technology for intra- and extracellular monitoring of cells.

Another important aspect to consider is the lack of focus in simplifying the monitoring of cell secretion for end-users. The technologies presented are growing in complexity, requiring laborious fluidic networks, special expertise for cell culture, and unconventional analytical equipment often accompanied by elevated costs. Considering the profile of researchers who will require these new technologies, mostly found in biological research laboratories and pharmaceutical industries, it is highly advisable for future research on cell monitoring technologies to make them compatible with conventional processes and equipment found in those settings, in order to bring new developments closer to day-to-day practice. This is particularly essential when considering that cell secretion is just one aspect of cell monitoring. Enabling the coupling of secretion monitoring with other standardized cell assays typically conducted using common equipment, such as immunocytochemistry, morphology, and proliferation optical monitoring, is a crucial aspect to take into account moving forward.

Finally, it is important to highlight that this area of research is still in a very early stage, and the amount of truly new knowledge acquired by these new approaches compared to conventional in vitro assays is still very limited. As the expectation grows regarding the implementation of novel systems in real pharmaceutical development, especially with the reduction of animal models in mind, what has been developed so far still needs to prove a real upgrade over the traditional, well-standardized in vitro methods. As some of the technologies presented in this review have showcased the capabilities to perform novel types of analysis not achievable through conventional methods, including the groundbreaking spatiotemporal correlation of cell secretion to a single secretory event and the novel approach to studying cell-cell paracrine communication [61, 62, 76, 85, 86, 94, 99], it is hoped that future work will not be limited to constant reinvention of the analytical methods, but also on the validation and application of the most promising technologies in real scenarios.

In conclusion, while significant advancements have been made in cell secretion monitoring within complex microenvironments, considerable challenges remain. The current focus highlights the need for more representative biological models as well as the requirement in validating new technologies in real scenarios. Addressing these areas will advance our understanding and application of cell secretion monitoring, ultimately enhancing biological research and pharmaceutical development.
